# Draft Genome Sequence of a Subarctic Humic Substance-Degrading Pseudomonad

**DOI:** 10.1128/genomeA.00070-12

**Published:** 2013-01-31

**Authors:** Ha Ju Park, Seung Chul Shin, Dockyu Kim

**Affiliations:** Division of Life Sciences, Korea Polar Research Institute, Incheon, Korea

## Abstract

The *Pseudomonas* sp. PAMC 26793 was isolated because of its high ability to degrade humic acids from a subarctic grassland in Alaska. We sequenced the PAMC 26793 genome to discover the genes for degradation of natural humic substances and to provide further information for the degradation process of soil bacteria in a low-temperature environment.

## GENOME ANNOUNCEMENT

Soil humic substances (HS), composed mainly of humic acids, are widely distributed in cold natural environments, such as alpine areas, the Arctic, and the Antarctic, and are known as an important fraction of soil organic carbon (1, 2, 3). HS, characterized by carboxylic acid and phenolic hydroxyl groups, are formed via the decomposition of plant materials, including lignocellulose, and the condensation of smaller molecules through biological and physical processes (4). Despite HS having been studied in many research fields, knowledge of the role played by microorganisms in forming and decomposing these substances is still insufficient. Current evidence suggests that soil bacteria play a critical role in the HS degradation process due to their prevalence, diversity, and catabolic versatility (3, 4). Until now, however, little has been known about the HS-degrading bacteria in cold environments, which led us to initiate the present study.

The genome of *Pseudomonas* sp. PAMC 26793 was analyzed using a 300-bp paired-end library (14,782,674 reads) with the Illumina HiSeq 2000 (Illumina, San Diego, CA) and a 7-kb paired-end library (77,008 reads) with the 454 GS FLX titanium system (Roche Diagnostics, Branford, CT). The reads were assembled into 58 contigs with Celera assembler 7.0 (5). The draft genome sequence of *Pseudomonas* sp. PAMC 26793 was approximately 6.7 Mb long with a G+C content of 59.8%. The resulting *N*_50_ size of contigs was 262,823 bp and the total coverage over the genome was 245-fold. Gene prediction and annotation using the Rapid Annotation using Subsystems Technology (RAST) pipeline (6) revealed 6,324 open reading frames (ORFs), 59 tRNA-encoding genes, and 14 rRNA genes in the draft genome.

### Nucleotide sequence accession numbers. 

This whole-genome shotgun project has been deposited at DDBJ/EMBL/GenBank under the accession no. AMXG00000000. The version described in this paper is the first version, AMXG01000000.
